# Diet Quality and Mental Health Status among Division 1 Female Collegiate Athletes during the COVID-19 Pandemic

**DOI:** 10.3390/ijerph182413377

**Published:** 2021-12-19

**Authors:** Natalie Christensen, Irene van Woerden, Nicki L. Aubuchon-Endsley, Pamela Fleckenstein, Janette Olsen, Cynthia Blanton

**Affiliations:** 1 Department of Nutrition and Dietetics, Idaho State University, Pocatello, ID 83201, USA; pamelafleckenstei@isu.edu (P.F.); cynthiablanton@isu.edu (C.B.); 2 Department of Community and Public Health, Idaho State University, Pocatello, ID 83201, USA; irenevanwoerden@isu.edu (I.v.W.); janetteolsen@isu.edu (J.O.); 3 Department of Psychology, The University of Tulsa, Tulsa, OK 74104, USA; aubucho@ostatemail.okstate.edu

**Keywords:** female, collegiate athletes, diet quality, mental health, Healthy Eating Index, COVID-19, nutrition, stress

## Abstract

The International Olympic Committee has identified mental health as a priority that significantly affects the physical health and safety of collegiate athletes. Interventions that improve diet quality have been shown to improve mental health in several populations. However, studies are needed to examine this relationship in female collegiate athletes, who have elevated risk of experiencing anxiety and depression symptoms, as well as dietary insufficiencies. In a quantitative, cross-sectional study, female student athletes at a U.S. university completed three mental health questionnaires: Depression Anxiety and Stress Scale (DASS-21), Athlete Psychological Strain Questionnaire (APSQ), and COVID Stress Scales (CSS). Each female athlete also completed a validated, web-based Diet History Questionnaire (DHQ-III) resulting in a Healthy Eating Index (HEI). Seventy-seven participants completed all survey information. HEI scores were consistently higher for athletes with poorer mental health. HEI scores were significantly positively associated with stress (*p* = 0.015), performance concerns (*p* = 0.048), CSS components of danger (*p* = 0.007), contamination (*p* = 0.006), and traumatic stress (*p* = 0.003). Although findings support statistically significant associations among dietary quality and mental health indicators, including broad symptom severity or stressors specific to athletics or COVID-19, these associations were in the opposite direction hypothesized. Possible reasons for results and suggestions for future research are discussed.

## 1. Introduction

Mental health is a well-recognized risk factor for poor physical health and safety, with more recent research supporting these associations in collegiate athletes [1,2]. Along with the National Collegiate Athletics Association (NCAA) and the International Olympic Committee (IOC), the emerging field of Nutritional Psychiatry is seeking to address diet as a contributor to mental health. Nutritional Psychiatry research has shown that there is potential in using diet to prevent and treat certain mental health disorders [3,4,5,6,7]. Additionally, dietary interventions have been shown to improve mental health in several populations [5,6,8]. However, studies are lacking in their examination of this relationship in female collegiate athletes, despite the fact that they may have greater risk of experiencing anxiety, depression, and dietary insufficiencies [2,9,10,11,12]. Moreover, the coronavirus disease of 2019 (COVID-19) pandemic has impacted the lives of collegiate athletes in unique ways, which may increase their risk for dietary and mental health difficulties [13,14,15].

Observational studies indicate a connection between diet quality and mental health symptoms [3,5,7,16,17]. For example, Rao et al. examined diet adequacy in a sample of participants with depression and concluded that nutrient intakes failed to reach recommended levels [18]. Rao et al. further suggested that poor food choices may contribute to the development of depression [18]. A systematic review and meta-analysis of community dwelling adults by Lai et al. included gathering results from 13 observational studies, linking higher diet quality, defined as a more nutrient-dense intake, with a decreased risk of depressive symptoms [19]. Another systematic review supporting the relationship between mental health difficulties and poor diet quality in children and adolescents was completed by O’Neil et al. [3]. Both reviews found consistent trends in the literature showing better mental health outcomes associated with higher diet quality as well as poorer mental health outcomes associated with poorer diet quality.

Many, but not all, investigations support a protective effect of high-quality diet on mental health. Kim et al. found that a low-quality dietary pattern was not significantly associated with depression in either men or women [20]. However, high-quality dietary pattern scores were inversely associated with depression in women, but not in men, suggesting a potential gender difference in this relationship. A systematic review and dose-response meta-analysis of prospective studies investigating the association of diet quality and depression found evidence that a higher-quality diet reduced the risk of the onset of depressive symptoms, but the authors stated that available evidence does not support firm conclusions that diet influences overall depression risk [21]. Lange et al. also discussed how the available evidence is inconclusive, but argued for the promotion of healthy eating patterns on a public health level due to the potentially important associations among dietary patterns, adiposity, inflammation and mental health [22].

Intervention studies provide further evidence supporting a role of nutrition in mental well-being. In adults experiencing depression, a randomized controlled trial conducted by Jacka et al. included measuring depressive symptoms and diet quality at the beginning and end of a 3-month nutrition intervention program [5]. They documented a significant improvement in depressive symptoms and diet quality in the nutrition intervention group compared with controls. Similarly, in multi-ethnic adults in a corporate setting, Agarwal et al. conducted a randomized controlled trial and found significant reductions in depression and anxiety in the diet intervention group compared to the control group [8]. In addition to these two studies, a meta-analysis by Firth et al. found 14 other randomized controlled trials, through March, of 2018 of dietary interventions reporting improvements in symptoms of depression and/or anxiety [6].

Depression and anxiety are noted as common comorbidities in individuals struggling with eating disorders [23,24]. Compared to non-athletes, male and female athletes are at higher risk of developing an eating disorder [24], with female collegiate athletes being identified as at higher risk for eating disorders, disordered eating, and relative energy deficiency than their male athlete counterparts [9,10,24,25,26,27,28,29]. Disordered eating and diagnosable eating disorders exist on a spectrum of eating behaviors. Disordered eating, or maladaptive eating behaviors that do not fit formal criteria for a diagnosable eating disorder in the Diagnostic and Statistical Manual of Mental Disorders [30], seem to be the most common with up to 84% of athletes reporting engagement in maladaptive eating and weight control behaviors such as restrictive eating, fasting, binge eating, purging, compulsive exercising, and taking weight loss supplements [24,31,32,33]. The percentage of athletes who meet full diagnostic criteria for an eating disorder ranges widely in the literature from 1.1% to 49.2% [9,24,29,33,34] with no one disorder being identified as most common. Popularity and perceived benefits of restrictive diets, such as vegan, gluten-free or intermittent fasting diets, also may encourage athletes to engage in eating behaviors that encourage disordered eating and increase eating disorder risk [35,36]. Restrictive dieting creates energy and nutrient deficiencies, which also increases risk for psychological impacts such as depression, anxiety, and stress [25,37,38]. Relative Energy Deficiency in Sport, or RED-S, is a term that refers to inadequate caloric intake resulting in low energy availability (LEA) and impaired physiological functioning that includes, but is not limited to, metabolic rate, menstrual function, bone health, cardiovascular health, and psychological health [37,39]. LEA can contribute to or be caused by impaired mental health [39].

Collegiate athletes demonstrate relatively high levels of stress, anxiety, and depression [1,2,11,12] that may be amenable to dietary interventions. Student athletes encounter stressors beyond maintaining high academic performance that may precipitate depression, including sports injury, competitive performance, and career termination [1]. Due to the unique difficulties faced by collegiate athletes in the United States, the NCAA implemented the GOALS (Growth, Opportunities, Aspirations, and Learning of Students) survey in 2006. This survey is given to a subset of collegiate athletes across the nation every 4 years. A study utilizing GOALS data revealed that student athletes increasingly struggle with mental health issues, such as depression, with about 30 percent self-reporting that they have been overwhelmed during the past month [40]. A recent study by Wolanin et al. affirms the GOALS study results in its findings that 27.3% of Division I student athletes report clinically significant symptoms of depression [41]. Rates of mental distress among student athletes appear to be higher in women compared to men [12,41]. Rice et al. found markedly higher rates of anxiety among female athletes than male athletes [42].

Compounding the high levels of stress in collegiate athletes are the effects of the COVID-19 pandemic. Buckley et al. identifies the COVID-19 pandemic environment as one that increases the risk of development of other behavioral health disorders (i.e., eating disorders), food preoccupation, and body dissatisfaction in athletes [14]. Additionally, Morrey et al. highlights the lack of control during the pandemic as a source of anxiety for athletes [43]. The NCAA Student-Athlete Well-Being Study [44] gathered surveys from over 60,000 collegiate student athletes in the United States in the Spring and Fall of 2020. The survey was taken by twice as many female athletes as male athletes within all three NCAA Divisions. The survey results revealed that student athletes were grappling with mental health issues such as feeling overwhelmed, lonely, anxious, hopeless, and sad. As many as 36% of female athletes identified COVID-19 concerns as factors that negatively affected their mental health. Similarly, Sanborn et al., in a study gathering data on the prevalence of COVID-19 anxiety among Division I student athletes, found that female athletes endorsed significantly higher levels of COVID-19-related anxiety, depression, and stress than male athletes [15].

Given the relatively higher prevalence of mental distress, disordered eating, and eating disorders in female collegiate athletes [9,24,27,28,45] and evidence showing a direct relationship between higher diet quality and lower rates of depression and anxiety [3,4,5,6,7,8], this study sought to examine the association between diet quality and mental health in this population. This study occurred during a peak period of COVID-19 restrictions and, therefore, includes the unique contribution of pandemic stressors to the findings. The experimental hypothesis stated that diet quality is inversely related to levels of mental distress in female collegiate athletes.

## 2. Materials and Methods

This study was conducted in accordance with the Declaration of Helsinki and the protocol was approved by the Institutional Review Board, Human Subjects Committee, protocol number IRB-FY2020-167. All participants provided their informed consent before beginning the study.

A quantitative, cross-sectional study design was chosen to examine if diet quality was related to mental health. An interdisciplinary team of professionals from a Division 1 university in the Mountain West of the United States was created utilizing faculty members in the Departments of Nutrition and Dietetics, Psychology, Counselling, Community and Public Health, and Athletics.

**Figure 1:**
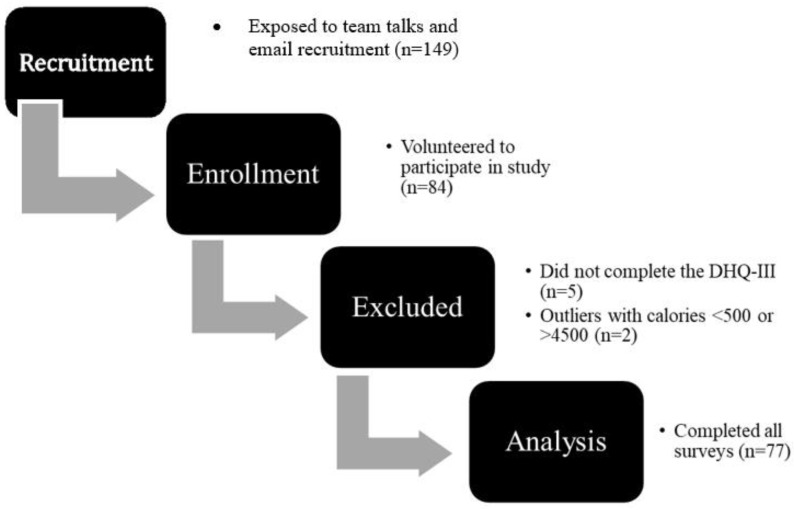
Flow Diagram of Participant Progress through Study Procedures.

The recruitment of participants took place through email and in-person discussions with head coaches of women’s sports teams, asking them to forward an email containing a brief description of the study purpose, methods, and inclusion criteria to their team members. The sports dietitian also met with individual teams and athletic administration to answer questions and discuss inclusion criteria. Participant flow through study procedures is shown in Figure 1.

### 2.1. Participants

#### Inclusion/Exclusion Criteria

For this study we limited our exclusion criteria in order to get the most variability or diversity represented in our primary study variables. Participant inclusion criteria was as follows: current collegiate athletes aged 18–25 years from the institution (an NCAA Division I university) who self-identified their gender as female. Female athletes were chosen given their relatively higher rates and unique risk factors for anxiety, stress, and depression compared to male athletes [12,41,42,46]. Female athletes also may be more open to discussing their mental health [47]. Considering that most students start right after high school, the minimum age was set at 18 years. If students had a “redshirt” year or a year in which they can practice with their team and receive financial aid, but they do not compete, the range up to 25 years of age would incorporate all years of competition eligibility. To keep the target population in mind, athletes with a prior diagnosis of depression or anxiety were eligible for the study. Female athletes struggling with eating disorders or restrictive eating, as well as increased fueling demands, were included in this study; thus, the range of energy consumption was broadened to include reported calorie intake as low as 500 kilocalories and as high as 4500 kilocalories.

### 2.2. Study Timeline

**Figure 2:**
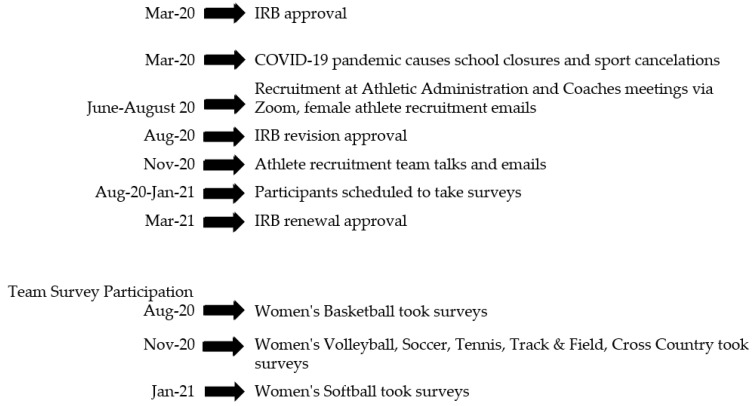
Study Timeline.

Data were collected from August 2020 to January 2021 from female collegiate athletes from multiple sports, including basketball, soccer, cross country, softball, tennis, and track and field. Women recruited from team talks and emails attended a specifically scheduled survey session for their team, where the investigators reviewed the study protocol and obtained informed consent from participants. All participants were informed of the study’s purpose and data collection methods, as well as confidentiality, prior to data collection. Consenting female student athletes were then guided into a computer lab and given access to five online questionnaires. Participants chose to begin with either the Diet History Questionnaire (DHQ-III) or the mental health questionnaires. Once the mental health questionnaires were electronically accessed, they were taken in order of the Athlete Psychological Strain Questionnaire (APSQ), the Depression Anxiety and Stress Scale (DASS-21), and then the COVID Stress Scales (CSS). The study timeline is depicted in Figure 2.

All participation in the study was voluntary, with steps taken to ensure confidentiality of information collected (e.g., de-identified data kept separately from signed consent forms). The registered sports dietitian was present at every survey session to answer any survey questions arising during the surveys. Clinical psychology and counselling research assistants were available during each study session to answer questions and support athletes in case of distress. A mental health resource sheet was created and on hand if a student athlete felt she needed more support. A licensed clinical psychologist was on-call during each survey session. Student information was de-identified using random number codes to protect participant information from inappropriate use. To avoid encroaching on participant rights, figures of authority (e.g., head coaches, athletic trainers, etc.) were not involved in data collection or analysis. Participants were given the option to request their mental health scores and/or their diet quality score on the Informed Consent form. Each athlete who made a request for her mental health score was contacted individually via email by the research team’s licensed clinical psychologist. Each athlete who requested to know her diet quality score was contacted via email by the registered dietitian.

### 2.3. Measures

#### 2.3.1. Depression Anxiety Stress Scale (DASS-21)

To assess levels of depression, anxiety, and stress in athletes, the commonly used DASS-21 questionnaire was selected. The DASS-21 is a three-part scale that measures symptom severity regarding depression and anxiety as well as levels of subjective stress. This is a validated tool that has been used with competitive athletes [13,48]. Vaughan et al., validated the DASS-21 among athletes within the context of COVID-19 [13]. High scores on the DASS-21 indicate elevated degrees of depression, anxiety, and stress. The DASS-21 consists of 21 questions and takes approximately 3–5 min to complete. Each of the sections contain seven items divided into subscales. The scores are added together for each section. The depression scale assesses symptoms of dysphoria, hopelessness, devaluation of life, self- deprecation, lack of interest/involvement, anhedonia, and inertia. The anxiety scale is used to quantify symptoms of autonomic arousal, skeletal muscle effects, situational anxiety, and subjective experience of anxious affect. The stress scales include questions regarding difficulty relaxing, nervous arousal, and being easily upset/agitated, irritable/over-reactive, and impatient [49].

#### 2.3.2. Athlete Psychological Strain Questionnaire (APSQ)

The APSQ is a mental health screening tool specifically for athletes. It provides important population-specific data beyond the DASS-21, such that when combined, the measures allow for a more comprehensive assessment of mental health among female collegiate athletes, which may be particularly important during the COVID-19 pandemic [13]. Rice et al. discuss the concept of psychological strain in athletes as a combination of perceived stress and difficulty coping [42,46]. This survey includes 10 questions asking how often an athlete feels certain emotions or experiences certain stressors like sport pressures or injury. An overall score, as well as scores for scales of Performance Concerns, Externalized Coping, and Self-Regulation Difficulties are calculated.

High scores on the APSQ indicate higher levels of athlete psychological strain. APSQ total scores are tiered into Moderate, High, and Very High ranges with cut off scores of 15–16, 17–19, and 20+, respectively [42]. For the purposes of this study, we utilized <20 as the range for Low and 20+ as the range for High. Based on the distribution of the responses, the Performance Concerns scale was given the cut off scores of <9 as Low and 9+ as high; the Externalized Coping scale was given the cut off scores of <3 as Low and 3+ as High; and the Self-Regulation Difficulties scale was given the cut off scores of <10 as Low and 10+ as High. These scores are meant to identify athletes who may need mental health support.

#### 2.3.3. COVID Stress Scale (CSS)

The third mental health survey given to participants was the CSS, which is a validated self-report instrument developed to better understand and assess distress related to COVID-19 [50]. Higher scores on the 36-item survey indicate higher COVID-19-related stress. The scale offers promise as a tool for better understanding distress associated with COVID-19 (and future pandemics) and for identifying people in need of mental health services [50]. In this study, the CSS was used as a tool to capture unique pandemic-related distress in female collegiate athletes and determine how it may be related to other important facets of mental health as well as nutrition. To keep the survey time no longer than 1.5 h, only four out of the six scales of the CSS were used, including Danger, Socioeconomic Consequences, Traumatic Stress, and Contamination, but not Xenophobia or Compulsive Checking. Responses for each of the four scales were dichotomized to low vs. high based on the distribution of the data.

#### 2.3.4. Diet History Questionnaire (DHQ-III)

The DHQ-III is an online questionnaire created by the National Cancer Institute for use in diet-related research [48,51]. The DHQ-III allows for assessment of diet quality using the Healthy Eating Index 2015 (HEI). The HEI scoring system has been validated in studies of collegiate athletes [52]. This study uses the food frequency questionnaire that gathers data from the past month with portion sizes. For this study, daily caloric intakes between 500 and 4500 were considered valid and retained for statistical analyses. The DHQ-III contains 135 questions on food and beverage consumption and 26 questions on dietary supplement use. Some of the main questions have additional clarification questions that could increase the number of questions to 263. Answers within question sets contribute to overall question clarity and a reduction in error.

### 2.4. Data Analysis

All analyses were conducted using R, Version 4.1.1. Descriptive statistics for all variables were quantified using mean and standard deviation values or subsample size and percentage. The association between each bivariate mental health subscale and the HEI score was examined using Welch’s nonparametric T-tests. Statistical significance was defined as *p* < 0.05.

## 3. Results

As depicted in the participant flow through study procedures chart in Figure 2, a total of 84 female student athletes consented to participate in this study. Five participants with an unfinished DHQ and unknown dietary quality were excluded. Two participants with reported daily kilocalorie intakes less than 500 were excluded with the assumption these could also reflect incomplete dietary reports. The total number of athletes utilized in statistical analysis was 77.

Participant characteristics are shown in Table 1. Participants were primarily unmarried (97%) and non-Hispanic White (68%) with a mean age of 19.3 years. The majority of participants stated that they were able to maintain their normal, pre-competition diet during quarantine (66%).

The DASS-21 results indicated that approximately half of the athletes had scores 8 or above for depression and stress, and 3 or above for anxiety (Table 2). Roughly half of the athletes were classified as having high/severe APSQ (50.6%; score 20 or above) and reported at least one item on the COVID-19 contamination fears scale from the CSS (53.2%; Table 2). Over ⅓ of participants reported at least one item on the CSS traumatic stress symptoms scale (35.1%).

**Figure 3:**
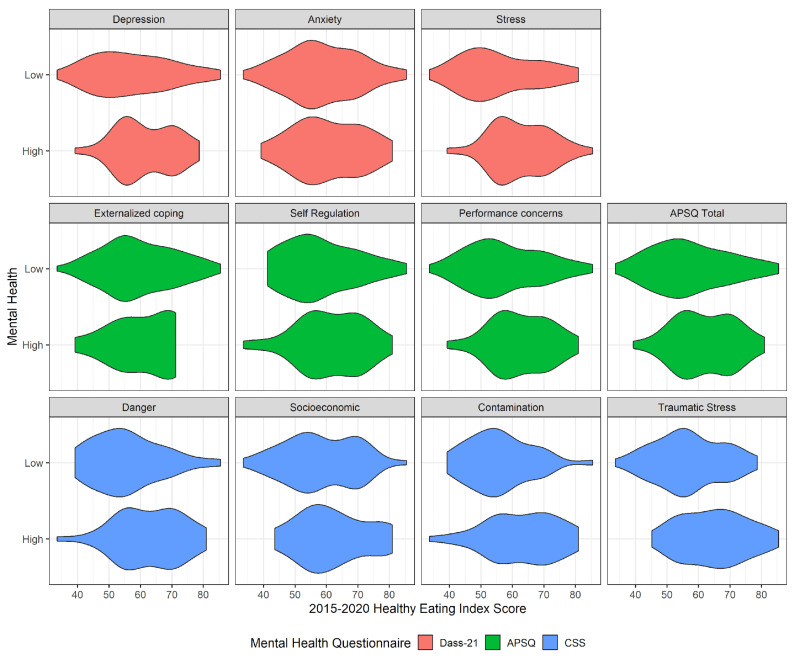
Visual Summary of the distribution of Healthy Eating Index Associations by Participant Mental Health variables.

Significant differences in HEI for several of the mental health measures were found (Table 2). For the DASS-21, a significant difference in HEI was found between participants who reported low and high stress (low stress = 56.1, high stress = 62.6, *p* = 0.015). For the APSQ, a significant difference was found for performance concerns (57.5 vs. 62.5; *p* = 0.048). Three of the four measures of CSS were also significant: danger (55.9 vs. 62.8; *p* = 0.007), contamination (56.0 vs. 63.0; *p* = 0.006), and traumatic stress (56.9 vs. 64.9; *p* = 0.003). While no significant differences for the other mental health measures were found, participants with worse mental health consistently had higher HEI scores than their counterparts. See Figure 3 for a visual summary of how HEI was associated with participant mental health. Specifically, Figure 3 is a violin plot demonstrating differences in the distribution of HEI scores between low and high scorers on each mental health index per questionnaire.

## 4. Discussion

The aim of this project was to investigate relationships between diet quality and mental health status among female collegiate athletes during the COVID-19 pandemic. Based on extant literature, we hypothesized that a higher-quality diet would be associated with lower rates of anxiety, stress, depression, athletic psychological strain, and COVID-19-related distress in female student athletes. Our main findings do not support our hypotheses. Results suggested that higher dietary quality is associated with *higher* levels of stress in female collegiate athletes. Similarly, female athletes with higher diet quality reported *greater* mental health struggles on the APSQ and CSS questionnaires. These findings are important in that they extend prior reports of psychological struggles unique to athletes and may be explained by considering emerging research within the context of unique risk factors for female collegiate athlete populations [13,28,53].

Namely, novel research supports that the COVID-19 pandemic has led to a worsening of food-body relationships among athletes, resulting in an increase in eating disorders and disordered eating nationwide [14]. This may be better understood by considering the concept of contextual body image in female athletes [54,55]. Contextual body image refers to the difference in an athlete’s body image within her sport as opposed to outside of her sport. For example, a tall, muscular female athlete may be proud and accepting of her body while on the volleyball court, but embarrassed and conscientious of her height and weight while at a social event unrelated to her sport. The COVID-19 transitions displaced athletes from their sports, thus, placing the socially acceptable body image at the forefront, which may have contributed to the increase of disordered eating and eating disorders [14,55]. These body image conflicts add yet another unique mental health struggle related to nutrition and diet quality for female collegiate athletes.

Specifically, athletes want to eat “healthy” for performance as well as for a socially acceptable body image. However, the concept of what is “healthy” differs from person to person and athletes are surrounded by conflicting nutrition information, which may put them at risk for restrictive diets and associated mental health difficulties [24,25,32,37,56]. Considering the important and unique factors of the female athlete population, as well as the current pandemic environment, it may be that the current finding of an association between higher diet quality and *greater* mental distress is due to female collegiate athletes who reacted to COVID-19 transitions by hyper-focusing on a socially encouraged body image and “healthy” eating, which may come in tandem with depression, stress and psychological strain.

Aside from unique body image factors, personality factors may also play a role in response bias to explain the unanticipated positive relationship supported between healthy eating and greater stress/distress. Becker et al. discusses research identifying athlete personality traits that lend well to competitive performance as well as to increasing risks of stress, anxiety, and disordered eating [2,10,32,57]. A significant proportion of athletes tend to be competitive and perfectionistic in many areas beyond sport, including academics [10]. How participants with these personality traits perceived the DHQ-III questionnaire could have affected their answers. For instance, in efforts to “get a good score” or demonstrate knowledge of higher quality food choices, these athletes could have chosen answers they knew to be correct, but were not true to life. The phenomenon of response bias may have significantly affected the diet quality scores; thus, alternative modalities of estimating dietary quality outside of self-report should be utilized in future research.

An outstanding question presented by the current findings pertains to the influence of the COVID-19 disruptions on diet. Was the impact of the pandemic so strong as to negate beneficial effects of high diet quality on levels of stress and depression? Alternatively, were higher diet quality scores a reflection of an attempt to control mental distress? Collegiate athletes experienced stress and uncertainty beyond normal financial and social stressors. The compounding effects of competition cancellations, constant COVID-19 testing, regular quarantines, isolations from family and friends, and academic struggles created a perfect storm for this young, vulnerable population, possibly encouraging unhealthy coping mechanisms.

In order to focus this study on overall diet quality and mental health, we did not gather anthropometric data. The body mass index (BMI) assessment was created to predict the potential of chronic diseases associated with obesity [58]. As a measure of weight relative to height, the BMI does not assess fat mass and fat-free mass (FFM). Its classification system was derived from the general population while many collegiate female athletes can be taller and more muscular. Therefore, BMI is not an accurate assessment of adiposity in this population with higher FFM [58,59,60]. In addition, the use of BMI in relation to eating disorders is somewhat problematic. Although identifying eating disorders is beyond the scope of this study, it is important to note that a low BMI measurement is associated with Anorexia Nervosa, yet individuals with Binge-Eating Disorder and Bulimia Nervosa may have normal or higher BMI measures [61]. It is difficult to unilaterally connect BMI with the presence of eating disorders. This, along with the body composition of female athletes, creates multiple confounding variables which negatively influences the accuracy and validity of BMI [27]. This study intentionally excluded the use of BMI because of these inherent problems.

This pilot study included several strengths, one being the interdisciplinary nature of the study as it brought the fields of psychology, counseling, public health, nutrition, and athletics together to explore new ideas for addressing mental health. Another strength is the inclusion of all women’s sports teams across 6 months of sport seasons. Further, researchers collected information on multiple components of mental health utilizing validated assessment tools. Limitations of the study should also be considered when interpreting findings, including the self-administration method of the questionnaires, which introduces bias. Another limitation is the possible bias introduced by the random nature of attention provided by research staff during questionnaire completion. That is, only participants that asked for clarification or assistance on questionnaires were provided with assistance by research staff. Collegiate athletes are also very busy juggling sport practice, classes, studying, homework, and employment, as well as community and social activities. The DHQ-III is a lengthy questionnaire requiring ~45 min to complete and participants may have rushed through the survey, resulting in inaccurate HEI scores. Further the combined time required to answer all questionnaires was longer than recommended [62]. We acknowledge that some evidence supports an association of menstrual phase and mental health and that the lack of these data is a limitation of this study [63,64,65]. It is also recognized that body composition data would strengthen future studies of the relationship between diet quality and mental health. Lastly, this pilot study did not include a large enough sample size to adequately guard against a Type II error (false negative) or for results to be considered representative of all female athletes. Future studies should include larger, more representative samples of female Division I athletes.

## 5. Conclusions

The current findings of a direct relationship between diet quality and mental distress levels in female collegiate athletes during COVID-19 suggest a need to re-evaluate the generalizability of the thought that higher diet quality is associated with better mental health. The impact of compounded life stressors on dietary behaviors deserves attention in investigations of nutrition as a means to optimize mental health.

## Figures and Tables

**Table 1:** Demographics and key variables for the female student athletes (*n* = 77).

Study Variable	
	mean (SD)
**Age**	19.3 (1.46)
	*n* (%)
**Year**	
First year (Freshman)	25 (32.91)
Second year (Sophomore)	14 (17.72)
Third year (Junior)	21 (26.58)
Fourth and Fifth year (Senior)	17 (22.78)
**Race**	
Non-Hispanic White	54 (68.35)
Non-Hispanic Black	3 (3.80)
Hispanic	7 (7.59)
Other	16 (20.25)
**Sport**	
Volleyball	12 (15.19)
Basketball	11 (13.92)
Softball	17 (21.52)
Tennis	8 (10.13)
Track and Field/Cross Country	10 (12.66)
Soccer	21 (26.58)
**Current Living Situation**	
On-Campus	28 (35.44)
Off-Campus	50 (63.29)
With Parents	1 (1.27)
**Married**	
Yes	2 (2.53)
No	75 (97.47)
**Living Situation from March 2020-July 2020**	
On-Campus	4 (5.06)
Off-Campus with roommates	16 (20.25)
At home with family	53 (67.09)
Other	6 (7.59)
**Working a job outside of sport and schooling**	
Yes	13 (16.46)
No	66 (83.54)
**Do you have an Eating Disorder?**	
Yes	2 (2.53)
No	75 (97.47)
**If yes, has your ED been diagnosed by a doctor?**	
Yes	
No	2 (100)
**Did COVID create financial hardship for you?**	
Yes	16 (20.25)
No	63 (79.75)
**Were you able to maintain your normal, pre-competition diet during quarantine?**	
Yes	52 (65.82)
No	27 (34.18)

**Table 2:** Association between Mental Health and Diet Quality for the 77 female athletes.

	*n* (%)	HEI, Mean (SD)	*p*-Value
DASS-21 *			
**Depression**			0.109
*Low (<8)*	36 (46.8)	57.5 (12.8)	
*High (8+)*	41 (53.2)	61.7 (9.5)	
**Anxiety**			0.511
*Low (<3)*	37 (48.1)	58.8 (11.8)	
*High (3+)*	40 (51.9)	60.6 (10.9)	
**Stress**			**0.015**
*Low (<8)*	34 (44.2)	56.1 (12.4)	
*High (8+)*	43 (55.8)	62.6 (9.5)	
APSQ *			
**Externalized Coping**			0.765
*Low (<3)*	64 (83.1)	59.6 (11.5)	
*High (3+)*	13 (16.9)	60.5 (10.2)	
**Self-Regulation**			0.218
*Low (<10)*	40 (51.9)	58.2 (11.6)	
*High (10+)*	37 (48.1)	61.4 (10.8)	
**Performance Concerns**			**0.048**
*Low (<9)*	43 (55.8)	57.5 (12.1)	
*High (9+)*	34 (44.2)	62.5 (9.6)	
**APSQ Total**			0.082
*Low (<20)*	38 (49.4)	57.5 (12.4)	
*High (20+)*	39 (50.6)	61.9 (9.7)	
CSS *			
**Danger**			**0.007**
*Low (<4)*	34 (44.2)	55.9 (11.1)	
*High (4+)*	43 (55.8)	62.8 (10.6)	
**Socioeconomic Consequences**			0.151
*Low (<2)*	45 (58.4)	58.2 (11.4)	
*High (2+)*	32 (41.6)	61.9 (10.9)	
**Contamination**			**0.006**
*Low (=0)*	36 (46.8)	56.0 (10.2)	
*High (1+)*	41 (53.2)	63.0 (11.3)	
**Traumatic Stress**			**0.003**
*Low (=0)*	50 (64.9)	56.9 (10.7)	
*High (1+)*	27 (35.1)	64.9 (10.5)	

* DASS-21 = Depression, Anxiety, and Stress Scale; APSQ = Athlete Psychological Strain Questionnaire; and CSS = COVID Stress Scales.

## Data Availability

The data presented in this study are available in Supplementary Table S1.

## Supplementary Materials

The following are available online at https://www.mdpi.com/article/10.3390/ijerph182413377/s1, Table S1, Dataset showing the HEI and mental health scores for the 77 female athletes.
