# Supplementary material for: Diet Quality and Mental Health Status among Division 1 Female Collegiate Athletes during the COVID-19 Pandemic
Source: Int J Environ Res Public Health. 2021 Dec 19;18(24):13377. doi: 10.3390/ijerph182413377 (PMC8703292; doi:10.3390/ijerph182413377)
Supplement: Supplementary file 1 [file ijerph-18-13377-s001.zip › ijerph-1464044-supplementary.pdf]

Table S1. Dataset showing the HEI and mental health scores for the 77 female athletes.

HEI	Kcal	Depression	Anxiety	Stress	Externalized_coping	Self_Regulation
58.8	661	1	1	1	0	0
78.74	1808.18	1	1	1	0	1
67.11	1946.65	0	1	1	0	0
69.16	1801.91	0	0	0	0	0
69.02	1782.67	1	0	1	0	1
80.96	2490.37	0	1	0	0	1
43.61	2823.26	0	0	0	0	0
47.81	3990.26	0	1	0	0	0
71.24	2254.49	1	1	1	0	0
49.02	731.51	0	0	0	0	0
70.77	2469.05	1	0	0	0	1
54.16	623.31	1	0	1	0	1
51.94	892.52	0	0	0	0	0
70.59	1045.41	1	1	1	0	1
52.2	3209.42	1	1	0	0	0
54.82	1208.87	1	0	1	1	1
72.42	596.83	1	1	1	0	1
68.67	1052.14	1	1	1	1	1
49.09	1135.68	0	1	0	0	1
56.44	1121.01	0	0	0	0	0
64.8	3466.52	1	1	1	0	1
59.88	1357.41	1	0	1	0	1
76.95	3642.78	0	1	1	0	1
71.21	876.84	1	1	1	1	0
78.21	1457.32	1	1	1	0	0
67.86	1167.51	0	0	0	0	0
53.6	1136.9	0	0	0	0	0
53.95	1711.01	1	1	1	0	1
62.99	2751.13	0	0	0	0	0
56.22	1869.59	1	1	1	0	1
78.68	727.49	1	0	0	0	0
54.98	1474.96	0	0	0	0	0
62.01	1641.34	0	1	1	0	1
55.4	2132.41	1	1	1	0	1
53.57	775.41	0	0	0	0	0
72.42	1865.91	0	0	0	0	0
68.42	624	1	1	1	1	1
52.96	924.19	1	1	1	0	0
45.08	1711.26	0	0	0	0	0
56.92	3580.77	0	1	1	0	0
63.93	1924.87	0	0	1	0	0
53.25	1389.52	1	1	1	0	1
44.65	639.22	0	1	0	0	0
69.7	1912.26	0	0	1	1	1
62.66	1865.44	1	1	1	0	1
62.98	714.96	1	1	1	0	1

74.35	1451.23	1	1	1	0	1
41.53	2536.46	0	0	0	0	0
55.94	1705.09	1	0	1	0	0
33.6	1569.67	0	0	0	0	1
45.21	1631.69	1	1	1	0	1
56.85	730.64	0	1	1	0	1
80.44	1679.4	0	0	0	0	0
39.26	2217.12	1	1	1	1	1
53.96	3339.67	0	0	0	0	1
85.49	2739.7	0	0	1	0	0
61.58	2974.92	0	1	0	0	0
50.14	3944.53	0	1	0	1	1
69.9	2262.57	1	1	1	1	1
56.34	1687.62	1	0	0	1	0
58.37	2854.99	1	1	1	1	1
59.55	2098.43	1	0	1	1	1
46.25	1525.6	0	0	0	0	0
53.91	2174.55	1	0	1	0	0
56.19	1309.98	1	1	1	0	1
52.63	2497.06	1	1	1	0	1
63.38	1538.69	0	0	0	0	0
41.34	1034.44	0	1	0	0	0
70.62	1724.76	1	1	0	0	1
68.75	1319.92	0	0	0	0	0
55.08	1572.65	1	0	1	0	0
63.98	1174.03	1	0	1	0	1
57.8	1837.98	1	1	1	0	0
49.64	1466.24	1	1	0	1	0
45.23	1907.88	0	0	0	0	0
70.9	2118.84	1	0	1	1	1
41.26	1267.46	0	0	0	0	0

Performance_concerns	APSQ_Total	Danger	Socioeconomic	Contamination	Traumatic_Stress
0	0	1	1	1	0
1	1	1	1	1	1
1	1	0	0	0	0
0	0	1	0	1	0
0	0	1	0	1	0
1	1	1	1	1	1
0	0	0	1	0	0
0	0	0	0	0	0
0	0	1	0	1	1
0	0	0	0	0	0
1	1	0	0	0	0
1	1	1	1	1	0
0	0	0	0	0	0
0	1	1	0	1	1
0	0	1	0	1	0
1	1	1	1	0	0
1	1	1	1	1	1
1	1	1	1	1	0
0	1	0	1	0	0
0	0	0	1	1	0
1	1	1	1	1	1
1	1	1	1	0	0
1	1	1	1	1	1
0	1	0	0	0	1
0	0	1	1	1	0
0	0	0	0	0	1
0	0	1	0	0	0
0	0	0	0	0	0
0	0	0	0	1	1
0	0	0	0	0	0
0	0	0	1	1	0
0	0	0	0	0	0
1	1	1	1	1	1
0	1	1	0	1	1
0	0	1	1	1	0
1	0	1	0	1	0
1	1	1	1	1	0
1	1	1	1	1	1
0	0	0	0	1	0
1	0	1	1	1	0
0	0	1	1	0	1
1	1	1	0	1	1
0	0	1	1	1	0
0	1	1	0	1	1
1	1	1	1	0	0
1	1	1	1	1	1

1	1	1	0	1	0
0	0	0	0	0	0
0	0	1	0	1	1
0	0	1	0	1	0
1	1	0	1	0	1
1	1	1	1	1	1
0	0	1	1	1	1
1	1	0	0	0	0
0	1	0	0	0	0
0	0	0	0	0	1
0	0	0	1	0	0
1	1	1	0	0	1
1	1	1	1	1	1
1	1	0	0	0	0
1	1	1	0	0	0
1	1	0	0	0	0
0	0	0	0	0	0
0	0	0	1	1	1
1	1	1	1	1	0
0	0	0	1	0	1
0	0	0	0	1	0
0	0	0	0	1	0
1	1	1	0	1	0
0	0	0	0	0	0
1	1	0	0	0	0
1	1	1	0	1	1
1	1	0	0	0	0
0	1	0	0	0	0
0	0	1	0	0	0
1	1	1	0	0	0
0	0	0	0	0	0
